# Wastewater-based genomic surveillance uncovers broader SARS-CoV-2 diversity and spatial transmission dynamics in Georgia, January 2023–July 2024

**DOI:** 10.1128/spectrum.01061-26

**Published:** 2026-09-29

**Authors:** Harrison Yu, Steven Woods, Carter O'Ryan, Jason Harrison, Cristina Meza, Hannah-Leigh Crawford, Hope Dishman, Bethany LaClair, Amanda Feldpausch, Ellen N. Kersh, Nandhakumar Balakrishnan, Tonia Parrott, Arunachalam Ramaiah

**Affiliations:** 1 Georgia Public Health Laboratory, Georgia Department of Public Health 37380, Decatur, Georgia, USA; 2 Epidemiology, Georgia Department of Public Health 37380, Atlanta, Georgia, USA; Barnard College, Columbia University, New York, New York, USA

**Keywords:** SARS-CoV-2, wastewater, clinical specimens, genomic surveillance, bioinformatics

## Abstract

Wastewater surveillance of severe acute respiratory syndrome coronavirus 2 (SARS-CoV-2) using next-generation sequencing (NGS) enables real-time monitoring of circulating and emerging variants, often providing earlier detection than clinical reporting while capturing presymptomatic, asymptomatic, and symptomatic infections. However, the correlation between lineage abundance in wastewater and clinical specimens from corresponding sewersheds in Georgia remains understudied. From January 2023 through July 2024, the Georgia Public Health Laboratory (GPHL) sequenced SARS-CoV-2 from 669 wastewater samples collected across 17 treatment facilities in 15 counties. These data were compared with 325 clinical specimens from corresponding sewersheds to assess early detection potential. Nucleic acids were extracted using the KingFisher Apex platform, followed by genomic library preparation and paired-end whole-genome sequencing on the Illumina NextSeq 2000. Variant calling and lineage abundance estimation using Freyja identified 2,218 SARS-CoV-2 lineages and sub-lineages, 1,769 in 2023, and 1,255 in 2024, with 806 co-circulating across both years. Clinical sequences spanned 135 lineages/sub-lineages across 15 clades, with >70% in clades 24A (27.4%), 23A (19.4%), 23F (13.5%), and 22E (10.2%). Predominant lineages included XBB.1.5 (11.4%), JN.1 (6.8%), and JN.1.4 (6.2%), reflecting broader clinical trends in Georgia. Clinical data also reflected distinct transmission patterns, including viral introduction and spread within and between counties. Overall, this study provides important insights into the introduction and dissemination of SARS-CoV-2 lineages in Georgia. Expanding wastewater surveillance to include other emerging and reemerging pathogens of public health importance will be particularly valuable during periods of low disease incidence, serving as an early warning system when clinical testing is limited and enabling timely public health responses.

**IMPORTANCE:**

Wastewater-based surveillance of SARS-CoV-2 provides a noninvasive, cost-effective, population-level method for detecting viral genetic material independent of clinical testing. Next-generation sequencing enables timely monitoring of circulating lineages by public health laboratories. The Georgia National Wastewater Surveillance System (GA-NWSS) established in 2021 through collaboration between the Georgia Department of Public Health (DPH) Epidemiology and the Georgia Public Health Laboratory (GPHL) collects weekly samples from 17 wastewater treatment facilities across 15 counties. From January 2023 through July 2024, GPHL sequenced 669 wastewater samples, identifying 2,218 SARS-CoV-2 lineages and sub-lineages, compared with 135 lineages detected in 325 clinical specimens from corresponding sewersheds. Spatial analysis highlights viral spread across state boundaries and within Georgia catchment areas, illustrating evolving transmission patterns. These results demonstrate that wastewater sequencing captures broader genomic diversity than clinical sampling alone, emphasizing its critical role in pathogen genomic surveillance and supporting data-driven public health response to SARS-CoV-2.

## OBSERVATION

Wastewater-based surveillance of SARS-CoV-2 is a noninvasive, cost-effective, population-level approach for the timely detection of viral genetic material in sewersheds, independent of clinical testing. Monitoring circulating SARS-CoV-2 lineages using next-generation sequencing (NGS) enables public health laboratories to conduct timely genomic surveillance. This approach, in collaboration with epidemiologists and supported by associated data on samples, populations, and disease trends, provides epidemiologic insights that enable early detection of emerging outbreaks, identification of novel variants, and monitoring of temporal trends in community transmission (1, 2). Furthermore, wastewater surveillance complements existing clinical systems by capturing presymptomatic, asymptomatic, and symptomatic infections.

In 2020, the Centers for Disease Control and Prevention (CDC) established the National Wastewater Surveillance System (NWSS) to support local-level tracking of SARS-CoV-2 transmission (2, 3). NWSS enables public health and academic laboratories the potential to detect emerging viral lineages ahead of clinical surveillance strategies and monitor genetic mutations associated with disease severity, diagnostic performance, vaccines, and therapeutics (3–5). In line with these efforts, the Georgia NWSS (GA-NWSS) program was established in 2021 as a collaboration between the Georgia Department of Public Health (DPH) Epidemiology and the Georgia Public Health Laboratory (GPHL). Through communication and partnerships with wastewater treatment facilities (WWTFs), Epidemiology onboarded 17 facilities across the state to contribute weekly samples for SARS-CoV-2 testing as part of the GA-NWSS program in conjunction with their routine compliance sampling. In addition to core SARS-CoV-2 testing, GPHL characterized lineage diversity and transmission trends in these wastewater samples using NGS and bioinformatics approaches and evaluated correlations between wastewater-derived lineages and those identified in clinical specimens from populations residing in geographic areas that feed into these sewersheds in Georgia.

**Fig 1:**
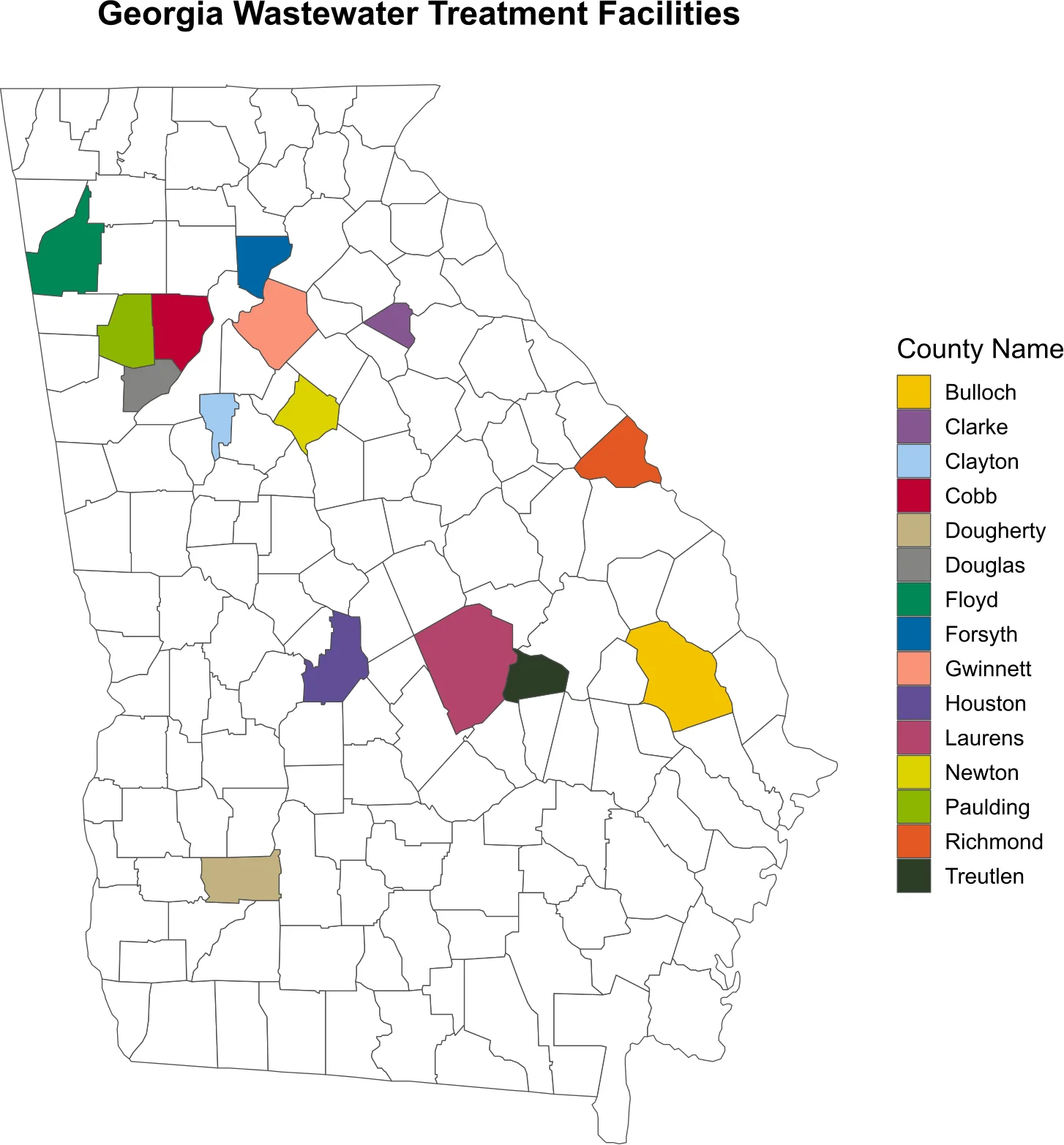
The Georgia map illustrates the 15 counties in which 17 WWTFs were located and from which wastewater samples were collected between January 2023 and July 2024.

After establishing partnerships between WWTFs, DPH Epidemiology, and GPHL in August 2021 for the GA-NWSS program, DPH Epidemiology worked with WWTFs to facilitate sample and metadata collection and transform laboratory data for assessing trends in SARS-CoV-2 concentrations across Georgia for public health use. As part of routine GA-NWSS program activities, GPHL received a total of 2,080 wastewater samples from 17 WWTFs (designated sites A–Q) across 15 counties in Georgia (Fig. 1) collected between January 2023 and July 2024. Nucleic acids were extracted using the KingFisher Apex system according to a standardized 10 mL concentration and extraction protocol. Samples were processed immediately upon receipt when possible or stored at 4°C as needed prior to extraction. Purified nucleic acids were stored at −80°C until further use. Libraries were prepared using ARTIC v4 primers, and paired-end whole-genome sequencing (WGS) was performed on the Illumina NextSeq 2000 platform. The same primer set was used throughout the study period to maintain methodological consistency across all samples. Raw reads were analyzed using a container-based Nextflow pipeline (https://github.com/yu-harrison/GPHL-Wastewater-Pipe) developed in-house at GPHL and run on the AWS HealthOmics environment, as detailed in Fig. S1. In addition, 325 SARS-CoV-2 genomes from clinical specimens obtained from patients residing within the corresponding wastewater catchment areas were identified in DPH State Electronic Notifiable Disease Surveillance System (SENDSS) using geocoded residential address information. These specimens were sequenced and analyzed using the Illumina DRAGEN COVID Lineage pipeline (Appendix). All raw reads from wastewater samples were submitted to the National Center for Biotechnology Information (NCBI) Sequence Read Archive under BioProject PRJNA1199543, while the 325 SARS-CoV-2 genomes from clinical specimens were deposited with the Global Initiative on Sharing All Influenza Data (GISAID) (Table S1). The DPH Ethics Committee determined that these genomic surveillance activities constituted public health surveillance and were not considered human subjects’ research.

**Fig 2:**
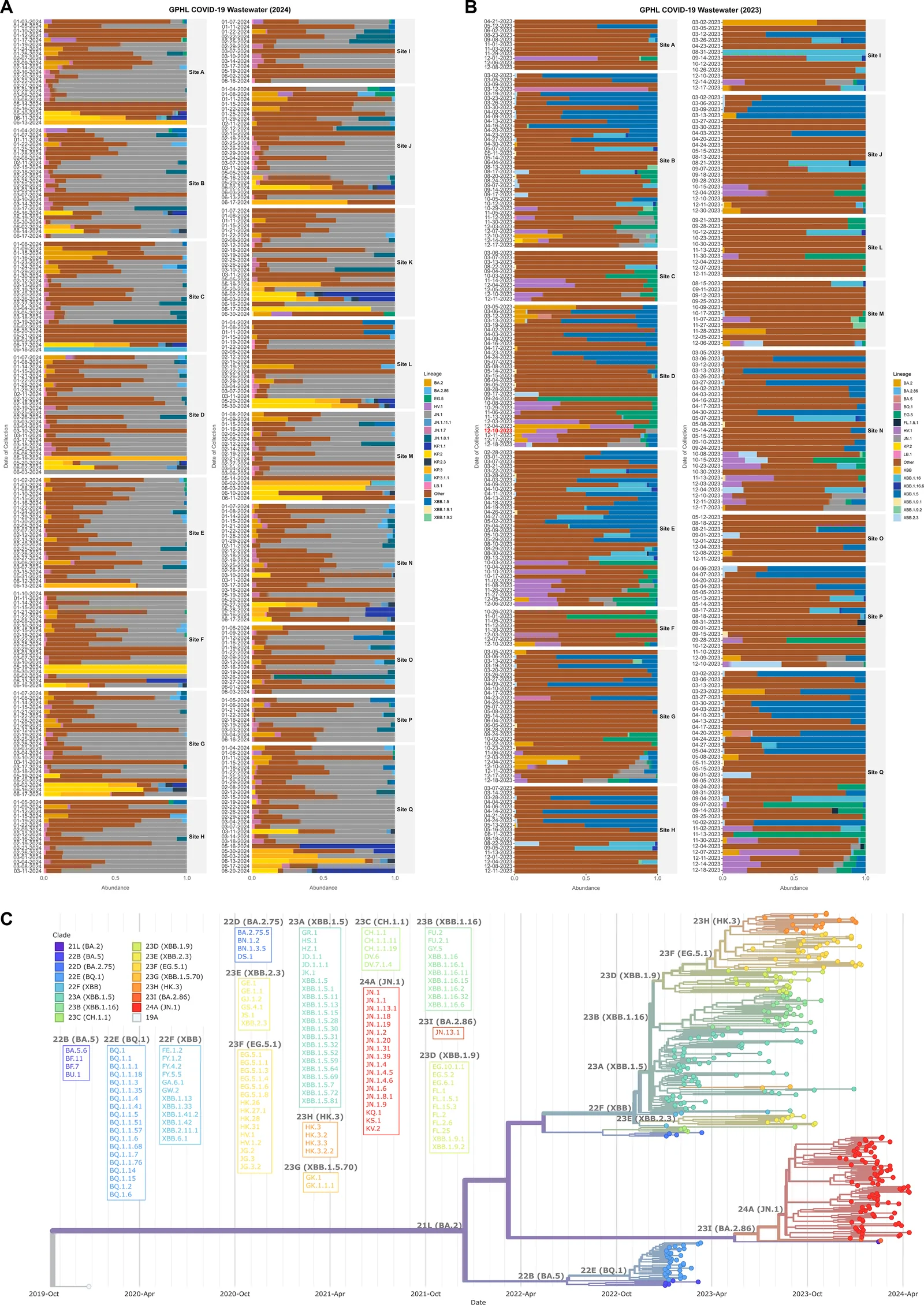
Classification and lineage assignment of SARS-CoV-2 sequences from wastewater and clinical specimens. The average relative abundance of accumulated SARS-CoV-2 lineages was assessed in 669 wastewater samples collected from (**A**) 16 sites in 2023 and (**B**) 17 sites in 2024. Lineages were grouped according to CDC NWSS guidelines. The average sequencing depth across all samples was 9,634×. (**C**) A Nextstrain-based phylogenetic representation was generated from 325 SARS-CoV-2 genomic sequences obtained from clinical specimens collected from patients residing within corresponding wastewater catchment areas between January 2023 and July 2024. Sequencing depth ranged from 621× to 42,339× (median, 8,231×; mean, 9,969×), and genomic coverage ranges ranged from 95.77 to 99.97% (median, 97.79%; mean, 97.87%). Variability in coverage was likely associated with sequence dropouts due to reduced performance of the ARTIC v4 primer set in specific regions, low amplification efficiency, or sequencing errors (6, 7).

Among the 2,080 wastewater samples, 725 (34.9%) were successfully sequenced for SARS-CoV-2. The relatively low sequencing success rate likely reflects the inherent challenges of wastewater samples, including variable viral loads, RNA degradation, and the presence of PCR inhibitors. Additionally, during the early phase of the GA-NWSS program, no pre-sequencing screening criteria or concentration thresholds were applied, which may have further contributed to the lower sequencing yield. Of the 725 sequenced samples, 669 (92.3%; 320 from 2023 and 349 from 2024) passed bioinformatics quality-control (QC) criteria and were included in downstream analyses. Lineage assignment for QC-controlled sequences from 17 sites identified 2,218 SARS-CoV-2 lineages/sub-lineages, including the following key emerging variants: BA.2, XBB.1.5, XBB.1.16, XBB.1.9, XBB.2.3, EG.5.1, XBB.1.5.70, HK.3, BA.2.86, JN.1, JN.1.11.1, and KP.3 (Fig. 2; Table S2). Interestingly, 1,769 and 1,255 lineages/sub-lineages were detected in 2023 and 2024, respectively, of which 806 were found to be co-circulating across both years (Table S2). These findings indicate that viral lineages detected in wastewater largely mirrored the SARS-CoV-2 genetic diversity circulating nationally at the time.

Further analysis showed that XBB.1.5 was the predominant lineage in wastewater samples from 14 counties in early 2023, but its prevalence began to decline around May 2023, highlighting that XBB.1.5 was the most frequently identified variant during that period (Fig. 2). Consistent with these observations, analysis of SARS-CoV-2 sequences from clinical specimens collected in Georgia during the study period revealed a similar trend, with XBB.1.5 representing a dominant lineage (17.5% of 6,079 genomes in GISAID; Nextclade 23A) (Table S3). To investigate the emergence of the JN.1 lineage, we compared wastewater sequence data across the 17 collection sites. JN.1 was first detected in wastewater on 8 November 2023 (Site E) and subsequently became an emerging and predominant lineage in wastewater throughout the study period (Fig. 2). This reflects a temporal shift in the dominant SARS-CoV-2 lineage, consistent with recent studies of United States clinical and wastewater samples (2, 8). Overall, the observed broader viral diversity and the emergence of new lineages in wastewater samples across sites were substantial and likely influenced by multiple factors, including population size, frequent travel, vaccination rates, large gatherings, and other epidemiologic variables.

To assess viral diversity in clinical samples from patients residing within the wastewater catchment areas of the studied counties, we sequenced and systematically analyzed 325 clinical specimens. These sequences were distributed across 135 lineages/sub-lineages belonging to 15 clades, with over 70% classified within clades 24A (27.4%), 23A (19.4%), 23F (13.5%), and 22E (10.2%) (Fig. 2C; Table S1). The predominant circulating lineages were XBB.1.5 (Omicron, 11.4%), JN.1 (Omicron, 6.8%), and JN.1.4 (Omicron, 6.2%), consistent with those in the broader clinical data set reported across Georgia (Table S3). Throughout the study period, no clades other than Omicron were detected, confirming that Omicron accounted for 100% of circulating viral lineages. To investigate localized transmission patterns, we performed a comprehensive phylogenetic analysis of clinical sequences from the collection sites alongside a global subsample of 4,179 SARS-CoV-2 sequences (9). This analysis demonstrated that clinical sequences from the same lineages (i.e., XBB.1.5 and JN.1) within community populations were more closely related to each other than to sequences from other regions, indicating circulation of genetically closely related strains and region-specific transmission trends (Fig. S2, Table S4).

To examine transmission dynamics of specific strains within and between the wastewater catchment area/counties, the 325 clinical sequences were further analyzed alongside the wastewater sequences. This comparative analysis revealed that the JN.1 lineage was detected in wastewater from Site-D on 10 December 2023, approximately 12 days before its first identification in a clinical specimen (EPI_ISL_18875445) from the same sewershed area on 24 December 2023 (Fig. 2; Table S5). This temporal difference underscores the value of wastewater surveillance for the early detection of novel variants before their appearance in clinical samples. Consistent with the wastewater data, this analysis also identified the JN.1 lineage as first emerging in November 2023 and persisting in the community until July 2024, marking the end of the study period.

Expanded comparative analysis of the clinical JN.1 strain EPI_ISL_18875445 (collection date: 22 December 2023) against 17.57 million existing SARS-CoV-2 genome sequences identified EPI_ISL_18532229 from Illinois (8 October 2023) as a closely related sequence. Within Georgia, EPI_ISL_18875456 (GPHL, 23 December 2023) and EPI_ISL_18808460 (CDC-GA, 28 December 2023) were also identified as related strains. Pairwise sequence alignment between EPI_ISL_18875445 and these three sequences revealed 0–2 nucleotide differences. These findings suggest that this specific JN.1 strain was possibly introduced into Georgia between October and December 2023 before it became widespread in local populations. Additionally, we identified the clinical XBB.1.5.7 strain EPI_ISL_16711194 (5 January 2023) from Site D as closely related to EPI_ISL_16816289 (9 January 2023) from Site P, confirming the transmission of a specific clinical strain between wastewater catchment areas/counties. Overall, these results highlight the potential for SARS-CoV-2 strain transmission both across state boundaries and within Georgia wastewater catchment areas, reflecting how viral dissemination successfully and dynamically unfolded in Georgia in the geographic areas monitored by the GA-NWSS program.

In summary, a total of 2,218 unique lineages/sub-lineages were detected across 669 wastewater samples when compared with 135 unique lineages identified in 325 clinical samples from individuals residing in the catchment areas. This more than 16-fold difference demonstrates that wastewater sequencing captures substantially greater SARS-CoV-2 genomic diversity than clinical surveillance alone. These findings highlight a significant clinical sequencing blind spot, as clinical sampling is inherently biased toward symptomatic individuals seeking care, whereas wastewater surveillance captures viral shedding from the broader community, including presymptomatic, asymptomatic, and symptomatic individuals.

Wastewater surveillance also enabled the early detection of the JN.1 lineage, identifying its presence 12 days before it was detected in clinical specimens. This lead time demonstrates the potential of wastewater surveillance to provide actionable early-warning signals that can support proactive public health responses, including healthcare resource planning and therapeutic distribution. Comparative genomic and phylogenetic analyses revealed the spatial and transmission dynamics of SARS-CoV-2 variants, illustrating patterns of viral introduction through domestic travel.

SARS-CoV-2 has transitioned to an endemic phase characterized by sustained circulation and periodic seasonal waves, similar to influenza and RSV. Although future variants may result in substantial increases in transmission, the magnitude of these outbreaks will depend on viral characteristics, immune escape, and the level of population immunity. Collectively, these findings underscore the importance of GA-NWSS and coordinated regional and national surveillance networks.

This study has several limitations. Occasional disruptions in sample collection, transfer, and trend analysis occurred because of holidays, shipping delays, and facility staffing limitations beyond the control of the DPH. These disruptions may have reduced temporal resolution during periods of peak transmission. To address these challenges, we subsequently conducted target-specific stability studies to systematically evaluate the effects of processing delays on sample integrity. Since the study period, additional optimization of sample processing workflows, RNA extraction protocols, primer sets, and pre-sequencing viral concentration thresholds have substantially improved sequencing performance, resulting in enhanced genome coverage and improved detection of newly emerging variants and sub-lineages.

In the future, the established wastewater genomic surveillance infrastructure could be expanded to monitor additional emerging and reemerging pathogens of public health importance, for example, measles and antimicrobial resistance (AMR) genes. To support this expansion beyond SARS-CoV-2, the GPHL is actively evaluating target-enrichment approaches (e.g., Twist hybrid capture) as well as shotgun and agnostic metagenomic sequencing methodologies. Pathogens of interest have been associated with recent or ongoing outbreaks or represent emerging health potential in Georgia and the United States; as a result, they represent important targets for enhanced public health surveillance. Wastewater surveillance, when integrated with existing epidemiological preparedness frameworks and sentinel surveillance systems, could be particularly valuable, potentially serving as an early warning system for emerging health threats, supporting epidemiological investigations, and enabling timely data-driven public health responses.

## Data Availability

The raw reads generated from wastewater samples were submitted to the NCBI Sequence Read Archive (SRA) under the accession range SRR31830283–SRR31831007, associated with BioProject PRJNA1199543. The assembled 325 SARS-CoV-2 genomes from clinical specimens were deposited with the GISAID database (Table S1).

## SUPPLEMENTAL MATERIAL

Supplemental material spectrum.01061-26-s0001.pdf Supplemental methods, associated references, legends for Tables S1 to S5, and Figs. S1 and S2.

Supplemental tables spectrum.01061-26-s0002.xlsx Tables S1 to S5.

ASM does not own the copyrights to Supplemental Material that may be linked to, or accessed through, an article. The authors have granted ASM a non-exclusive, world-wide license to publish the Supplemental Material files. Please contact the corresponding author directly for reuse.
