# Supplementary material for: Wastewater-based genomic surveillance uncovers broader SARS-CoV-2 diversity and spatial transmission dynamics in Georgia, January 2023–July 2024
Source: Microbiol Spectr. 2026 Sep 29;14(11):e01061-26. doi: 10.1128/spectrum.01061-26 (PMC13729166; doi:10.1128/spectrum.01061-26)
Supplement: Supplementary file 1 [file spectrum.01061-26-s0001.pdf]

Wastewater-Based Genomic Surveillance Uncovers Broader SARS-CoV-2 Diversity and Spatial Transmission Dynamics in Georgia, January 2023-July 2024

Harrison Yu[1], Steven Woods[1], Carter O'Ryan[1], Jason Harrison[1], Cristina Meza[2], Hannah-Leigh Crawford[2], Hope Dishman[2], Bethany LaClair[2], Amanda Feldpausch[2], Ellen N. Kersh[1], Nandhakumar Balakrishnan[1], Tonia Parrott[1], Arunachalam Ramaiah[1, *]

SUPPLEMENTAL TEXT

MATERIALS AND METHODS

Sample collection

The Georgia Department of Public Health (DPH) National Wastewater Surveillance System (GA NWSS) was established in August 2021. Following its establishment, the program required approximately eight months to build partnerships among wastewater treatment facilities (WWTFs), DPH Epidemiology, and the Georgia Public Health Laboratory (GPHL) to support wastewater sample collection for monitoring circulating SARS-CoV-2 lineages and trends in Georgia for public health use. As part of this effort, epidemiologists worked closely with WWTFs to facilitate sample and metadata collection. Wastewater samples are collected by WWTF staff on a schedule consistent with the site's existing compliance testing sample collection. Between January 2023 and July 2024, a total of 2,080 wastewater samples were collected from 17 wastewater treatment facilities (designated Sites A-Q) across 15 Georgia counties (Figure 1) using a standardized collection kit that included 3-4 ice packs, two plastic bags, and two absorbent pads. Two 50 mL samples were collected per site per week and shipped to the GPHL for further analysis. Any variations or gaps in wastewater sampling were due to factors such as holidays, logistical constraints, staffing shortages, and other onsite or shipping issues, which affected sample transfer to GPHL and hindered timing of comprehensive trend analysis.

Processing and initial testing of samples

The received wastewater samples were considered acceptable for processing if they were intact and no more than one week old; samples that were damaged, leaked, or improperly labeled were excluded. Samples were processed on the same day or refrigerated at 4°C if delayed. Storage at

4°C for up to one week did not significantly affect target recovery. To improve downstream processing and reduce potential inhibitors, viral particles were concentrated from 10 mL of wastewater using Ceres Nanotrap particles. Viral RNA was then extracted using the Thermo RNA extraction kit on the KingFisher Apex magnetic extraction platform and stored at −80 °C. Target detection and quantification were performed using the Qiagen Digital PCR platform with the SARS-CoV-2 Wastewater Surveillance Multiplexed Assay Kit.

Sequencing and bioinformatics analysis of SARS-CoV-2 in wastewater samples

Sequencing was performed for 725 samples using a modified Illumina COVIDSeq Test Protocol. cDNA amplification was conducted using Illumina COVIDSeq v4 Primer Pools, followed by tagmentation and PCR amplification. The resulting amplicons underwent post-tagmentation cleanup, library pooling, and normalization. Libraries were quantified using Qubit fluorometry to determine DNA concentration, and average library size (bp) was assessed using the Agilent TapeStation 2200. These measurements were used to calculate library DNA molarity. Paired-end libraries were diluted and sequenced (2 × 150 bp) on the Illumina NextSeq 2000 platform (https://www.illumina.com) using the NextSeq 1000/2000 P2 Reagent Kit.

Sequencing data from wastewater samples were processed using a container-based Nextflow in-house pipeline (https://github.com/yu-harrison/GPHL-Wastewater-Pipe) deployed on the AWS HealthOmics platform (Figure S1). Initial quality control (QC) was performed with FastQC, followed by removal of human-derived reads using SRA Reads Scrubber (1). Adapter sequences were trimmed using FastP with default parameters (2). Filtered reads were aligned to the SARS-CoV-2 reference genome (MN908947.3) using the BWA-MEM aligner (3). Read filtering and sorting were conducted with samtools (4), and ARTIC v4 primer sequences were trimmed using iVar with a minimum quality threshold of 20 (−q 20) and a minimum retained read length of 50 bp (−m 50).

Variant calling and lineage abundance estimation were performed using Freyja v1.5.1 (5). Usher barcodes and curated lineage files were obtained from the Freyja GitHub repository (https://github.com/andersen-lab/Freyja-data) on July 17, 2024, and used as inputs for Freyja demix. Lineage abundance estimation was successful for 669 of the 725 samples; the remaining samples had insufficient sequencing depth for analysis. The workflow utilized the following Docker images from the StaPH-B repository (https://github.com/StaPH-B/docker-builds): FastQC v0.12.1, FastP v0.23.4, BWA v0.7.17, iVar v1.4.2, Freyja v1.5.1, and SRA Human Scrubber v2.2.1.

Sequencing and bioinformatics analysis of SARS-CoV-2 in clinical specimens

Clinical specimens from nasopharyngeal swabs were stored in viral transport media at -80°C. For sample selection, the validated Thermo Fisher TaqMan™ Gene Expression Capillary real-time PCR assay was used to detect SARS-CoV-2-postivie specimens. The nucleic acid extraction method was validated and automated with a liquid handler instrument DNA quantity and quality were assessed using a Qubit Fluorometer and a Nanodrop spectrophotometer. The quality-controlled RNA was used for library preparation with the Illumina DNA Prep Kit. Sequencing was performed specimens with *Ct* value <30 on an Illumina NextSeq1000 and NextSeq2000 (Illumina, San Diego, CA) platform using NextSeq1000/2000 P2 200-cycle cartridges and flow cells, targeting ≥100× average genomic depth with 150 bp paired-end reads.

Paired-end raw data from the NextSeq 1000/2000 platform were assembled using the Illumina DRAGEN COVID Lineage pipeline (v3.5.13), which employs a customized version of the DRAGEN DNA pipeline for k-mer–based detection of SARS-CoV-2. Reads were aligned to the Wuhan Hu-1 reference genome for variant calling, generating consensus genome sequences. The resulting sequences were automatically submitted to GISAID using a custom Python script.

The clade and lineage assignment for each of 325 SARS-CoV-2 sequences were uniformly performed using Nextclade Pango Classifier in Nextclade CLI version 3.18.1 (6). Auspice 2.67.0 (7) was used to visualize the phylogenetic tree for these 325 sequences independently as well as with global sub-dataset. Closely related genomes for representative clinical strains were identified from the EpiCoV database using AudacityInstant v5.1.0 (8). Mutational analysis was conducted through multiple sequence alignment with Clustal Omega (9), followed by variant processing using msa2vcf, with genomic coordinates referenced to the Wuhan Hu-1 strain.

The SARS-CoV-2 genomes from 325 clinical specimens submitted by healthcare providers and/or laboratories for respiratory surveillance were obtained as part of DPH's routine notifiable disease surveillance. These specimens, collected from patients residing within the 17 corresponding wastewater catchment areas, were identified using geocoded residential addresses data from the DPH State Electronic Notifiable Disease Surveillance System (SENDSS).

Clinical specimens used in this study were received in accordance with Georgia Department of Public Health (DPH) Institutional Biosafety Committee (IBC) guidelines. The Ethics Committee determined that these genomic surveillance activities constituted public health surveillance and did not raise ethical concerns.

REFERENCES

1. Docker Hub. NCBI sra-human-scrubber Docker image. https://hub.docker.com/r/ncbi/sra-human-scrubber.
2. Shifu C. fastp 1.0: An ultra-fast all-round tool for FASTQ data quality control and preprocessing. iMeta 2025. https://doi.org/10.1002/imt2.107
3. Li H, Durbin R. Fast and accurate short read alignment with Burrows-Wheeler Transform (2009). Bioinformatics, 25:1754-60. https://doi.org/10.1093/bioinformatics/btp324
4. Danecek P, Bonfield J, Liddle J, Marshall J, Ohan V, Pollard MO, et al. Twelve years of SAMtools and BCFtools: GigaScience, Volume 10, Issue 2, February 2021, giab008, https://doi.org/10.1093/gigascience/giab008
5. Karthikeyan S, Levy JI, De Hoff P, Humphrey G, Birmingham A, Jepsen K, et al. Wastewater sequencing reveals early cryptic SARS-CoV-2 variant transmission. Nature. 2022;609:101–8.
6. Aksamentov I, Roemer C, Hodcroft EB, Neher RA. Nextclade: clade assignment, mutation calling and quality control for viral genomes. J Open Source Softw. 2021;6(67), 3773, https://doi.org/10.21105/joss.03773
7. Bedford T, Neher R, Aksamentov I, Andrews K, Change J, Hadfield J, et al. Nextstrain: real-time tracking of pathogen evolution, Bioinformatics (2018), https://doi.org/10.1093/bioinformatics/bty407
8. Khare S, et al. GISAID's Role in Pandemic Response. China CDC Weekly (2021), 3(49): 1049-1051, http://dx.doi.org/10.46234/ccdcw2021.255
9. Sievers F, Higgins DG, Clustal Omega, Current Protocols in Bioinformatics (2013), https://dx.doi.org/10.1002/0471250953.bi0313s48

Supplemental Figures and Tables

Figure S1 - Bioinformatics workflow for analyzing SARS-CoV-2 in wastewater samples. Raw sequencing reads were processed using a container-based Nextflow pipeline deployed on the AWS HealthOmics environment. Sequencing quality control was performed with FastQC, followed by removal of human-derived reads using SRA Reads Scrubber. Adapter trimming was conducted with FastP, and filtered reads were aligned to the SARS-CoV-2 reference genome (MN908947.3) using the BWA-MEM aligner. Variant calling and estimation of SARS-CoV-2 lineage abundance were performed using Freyja v1.5.1.

Data and sequence file types: FASTQ (Format for Advanced Sequencing Technology Quality) files contain raw sequencing reads with base quality scores, while FASTA (Fast-All) files contain sequences without quality data. BAM (Binary Alignment/Map) is a compressed binary file of aligned sequencing reads and BAI (Binary Alignment Index) is its index file for fast access; TSV is a tab-separated value - text table, BED (Browser Extensible Data) defines genomic coordinates, GFF (General Feature Format) annotates genomic features, and HTML (HyperText Markup Language) is a markup format for structuring and displaying web pages.

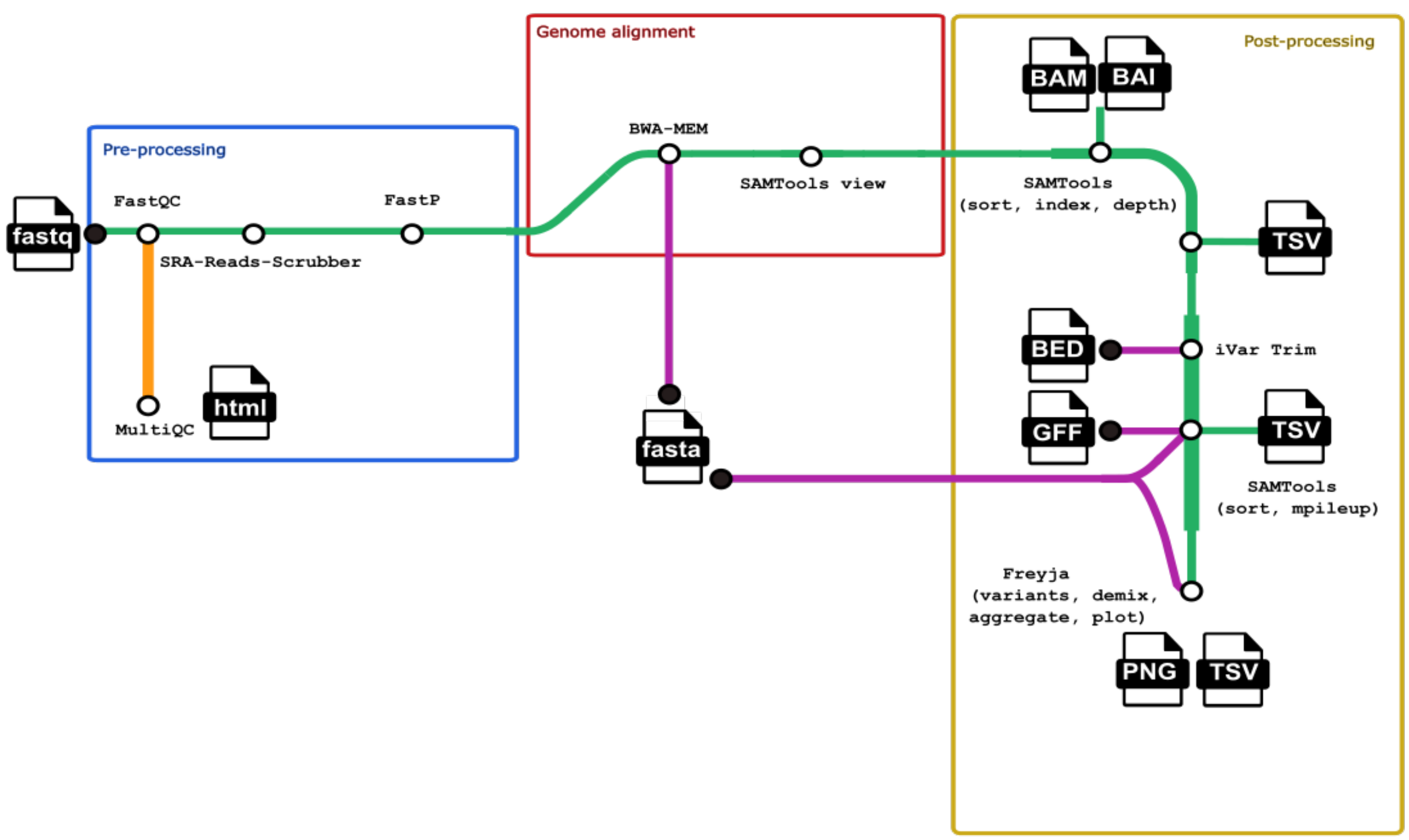

Figure S2 - Identification of clusters of genetically closely related clinical sequences from Georgia wastewater catchment areas. (**A**) Phylogenetic analysis of 325 SARS-CoV-2 sequences from Georgia, analyzed in the context of a global subsample of 4,179 sequences (Nextstrain; accessed January 20, 2026; see Table S4 for accession numbers and additional details). Prior to tree construction, all sequences were aligned to the reference strain Wuhan-Hu-1/2019. (**B**) and (**C**) Expanded views of representative phylogenetic clusters of Georgia XBB.1.5 and JN.1 sequences, respectively.

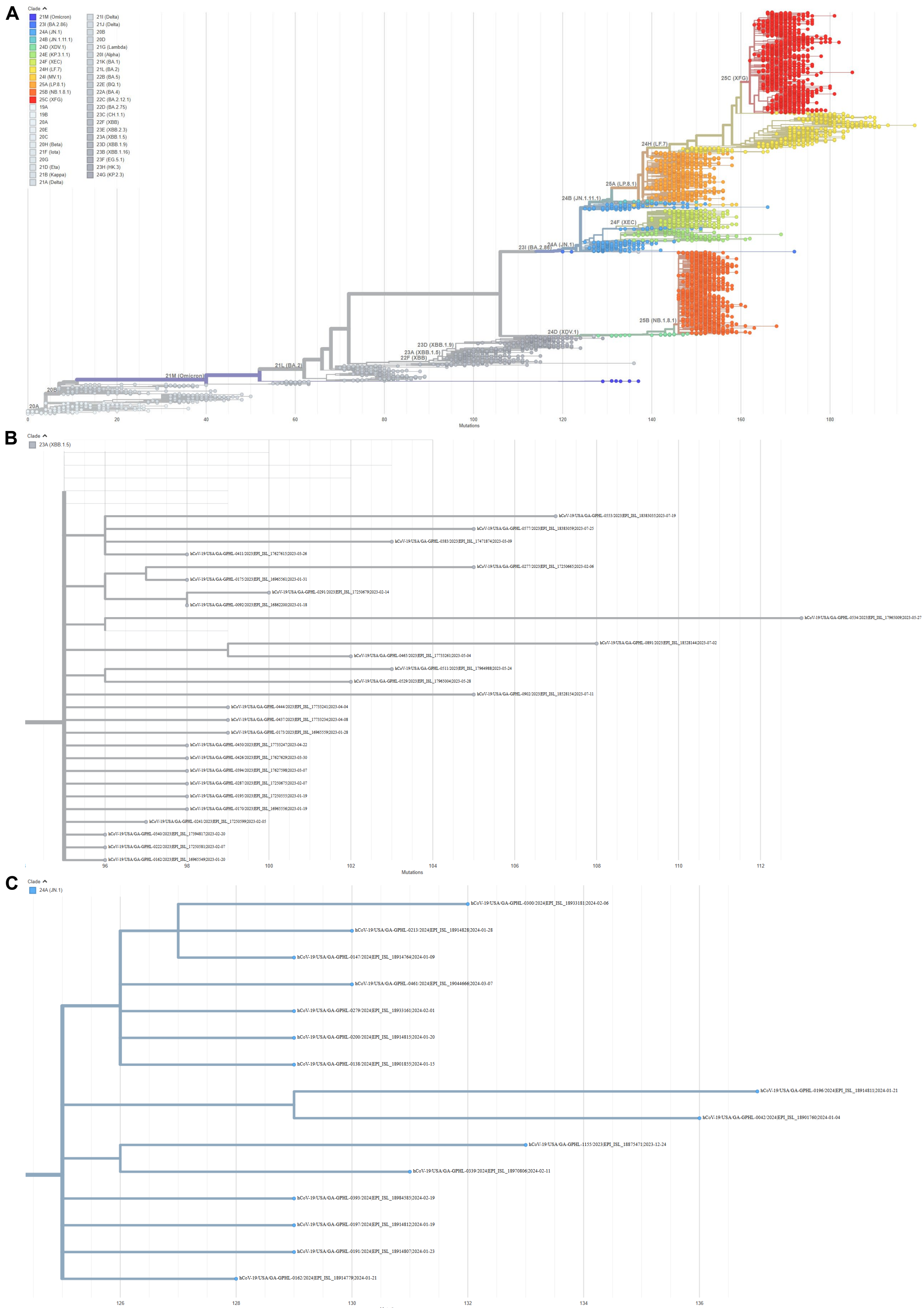
A
Clade
21M (Omicron)
23I (BA.2.86)
24A (JN.1)
24B (JN.1.11.1)
24D (XDV.1)
24E (KP.3.1.1)
24F (XEC)
24H (LF.7)
24I (MV.1)
25A (LP.8.1)
25B (NB.1.8.1)
25C (XFG)
19A
19B
20A
20E
20C
20H (Beta)
21F (Iota)
20G
21D (Eta)
21B (Kappa)
21A (Delta)
21I (Delta)
21J (Delta)
20B
20D
21G (Lambda)
20I (Alpha)
21K (BA.1)
21L (BA.2)
22B (BA.5)
22E (BQ.1)
22A (BA.4)
22C (BA.2.12.1)
22D (BA.2.75)
23C (CH.1.1)
22F (XBB)
23E (XBB.2.3)
23A (XBB.1.5)
23D (XBB.1.9)
23B (XBB.1.16)
23F (EG.5.1)
23H (HK.3)
24G (KP.2.3)
25C (XFG)
24H (LF.7)
25A (LP.8.1)
24B (JN.1.11.1)
24F (XEC)
23I (BA.2.86)
24A (JN.1)
25B (NB.1.8.1)
24D (XDV.1)
23D (XBB.1.9)
23A (XBB.1.5)
22F (XBB)
21L (BA.2)
21M (Omicron)
20B
20A
0
20
40
60
80
100
120
140
160
180
Mutations
B
Clade
23A (XBB.1.5)
hCoV-19/USA/GA-GPHL-0553/2023|EPI_ISL_18383035|2023-07-19
hCoV-19/USA/GA-GPHL-0577/2023|EPI_ISL_18383059|2023-07-25
hCoV-19/USA/GA-GPHL-0383/2023|EPI_ISL_17471874|2023-03-09
hCoV-19/USA/GA-GPHL-0411/2023|EPI_ISL_17627615|2023-03-26
hCoV-19/USA/GA-GPHL-0277/2023|EPI_ISL_17250665|2023-02-06
hCoV-19/USA/GA-GPHL-0175/2023|EPI_ISL_16965561|2023-01-31
hCoV-19/USA/GA-GPHL-0291/2023|EPI_ISL_17250679|2023-02-14
hCoV-19/USA/GA-GPHL-0092/2023|EPI_ISL_16862200|2023-01-18
hCoV-19/USA/GA-GPHL-0534/2023|EPI_ISL_17965009|2023-05-27
hCoV-19/USA/GA-GPHL-0891/2023|EPI_ISL_18528144|2023-07-02
hCoV-19/USA/GA-GPHL-0465/2023|EPI_ISL_17733261|2023-05-04
hCoV-19/USA/GA-GPHL-0511/2023|EPI_ISL_17964988|2023-05-24
hCoV-19/USA/GA-GPHL-0529/2023|EPI_ISL_17965004|2023-05-28
hCoV-19/USA/GA-GPHL-0902/2023|EPI_ISL_18528154|2023-07-11
hCoV-19/USA/GA-GPHL-0444/2023|EPI_ISL_17733241|2023-04-04
hCoV-19/USA/GA-GPHL-0437/2023|EPI_ISL_17733234|2023-04-08
hCoV-19/USA/GA-GPHL-0173/2023|EPI_ISL_16965559|2023-01-28
hCoV-19/USA/GA-GPHL-0450/2023|EPI_ISL_17733247|2023-04-22
hCoV-19/USA/GA-GPHL-0426/2023|EPI_ISL_17627629|2023-03-30
hCoV-19/USA/GA-GPHL-0394/2023|EPI_ISL_17627598|2023-03-07
hCoV-19/USA/GA-GPHL-0287/2023|EPI_ISL_17250675|2023-02-07
hCoV-19/USA/GA-GPHL-0195/2023|EPI_ISL_17250555|2023-01-19
hCoV-19/USA/GA-GPHL-0170/2023|EPI_ISL_16965556|2023-01-19
hCoV-19/USA/GA-GPHL-0241/2023|EPI_ISL_17250599|2023-02-05
hCoV-19/USA/GA-GPHL-0340/2023|EPI_ISL_17394817|2023-02-20
hCoV-19/USA/GA-GPHL-0222/2023|EPI_ISL_17250581|2023-02-07
hCoV-19/USA/GA-GPHL-0162/2023|EPI_ISL_16965549|2023-01-20
96
98
100
102
104
106
108
110
112
Mutations
C
Clade
24A (JN.1)
hCoV-19/USA/GA-GPHL-0300/2024|EPI_ISL_18933181|2024-02-06
hCoV-19/USA/GA-GPHL-0213/2024|EPI_ISL_18914828|2024-01-28
hCoV-19/USA/GA-GPHL-0147/2024|EPI_ISL_18914764|2024-01-09
hCoV-19/USA/GA-GPHL-0461/2024|EPI_ISL_19044666|2024-03-07
hCoV-19/USA/GA-GPHL-0279/2024|EPI_ISL_18933161|2024-02-01
hCoV-19/USA/GA-GPHL-0200/2024|EPI_ISL_18914815|2024-01-20
hCoV-19/USA/GA-GPHL-0138/2024|EPI_ISL_18901855|2024-01-15
hCoV-19/USA/GA-GPHL-0196/2024|EPI_ISL_18914811|2024-01-21
hCoV-19/USA/GA-GPHL-0042/2024|EPI_ISL_18901760|2024-01-04
hCoV-19/USA/GA-GPHL-1155/2023|EPI_ISL_18875471|2023-12-24
hCoV-19/USA/GA-GPHL-0339/2024|EPI_ISL_18970806|2024-02-11
hCoV-19/USA/GA-GPHL-0393/2024|EPI_ISL_18984585|2024-02-19
hCoV-19/USA/GA-GPHL-0197/2024|EPI_ISL_18914812|2024-01-19
hCoV-19/USA/GA-GPHL-0191/2024|EPI_ISL_18914807|2024-01-23
hCoV-19/USA/GA-GPHL-0162/2024|EPI_ISL_18914779|2024-01-21
126
128
130
132
134
136
Mutations

Table S1 – Details of 325 SARS-CoV-2 sequences from clinical samples of patients residing within wastewater catchment areas between January 2023 and July 2024. *Please refer to the Table_S1 sheet in the Excel file.*

Table S2 – Distribution and frequency of SARS-CoV-2 lineages detected in wastewater samples collected from 16 sites in 2023 and 17 sites in 2024. *Please refer to the Table_S2 sheet in the Excel file.*

Table S3 - Metadata for 6,079 genomes retrieved from the GISAID database, collected between January 2023 and July 2024. *Please refer to the Table_S3 sheet in the Excel file.*

Table S4 - **Acknowledgment of Data Contributors.** The hyperlinks provided give access to data contributor information and GISAID EpiCoV accession IDs for (A) 325 SARS-CoV-2 genomes generated by GPHL, (B) 6,079 SARS-CoV-2 genomes from Georgia, and (C) 4,179 SARS-CoV-2 genome sequences subsampled from a global dataset. *Please refer to the Table_S4 sheet in the Excel file.*

Table S5 – Details of closely related SARS-CoV-2 genomes of the representative JN.1 clinical strain from patient residing in the wastewater catchment areas. *Please refer to the Table_S5 sheet in the Excel file.*
